# Global Proteomic Analysis of Brain Tissues in Transient Ischemia Brain Damage in Rats

**DOI:** 10.3390/ijms160611873

**Published:** 2015-05-26

**Authors:** Jiann-Hwa Chen, Hsing-Chun Kuo, Kam-Fai Lee, Tung-Hu Tsai

**Affiliations:** 1 Institute of Traditional Medicine, School of Medicine, National Yang-Ming University, Taipei 112, Taiwan; E-Mail: chenjiannhwa@yahoo.com.tw; 2 School of Medicine, Fu-Jen Catholic University, Taipei 112, Taiwan; 3 Department of Emergency Medicine, Cathay General Hospital, Taipei 112, Taiwan; 4 Department of Nursing, Chang Gung University of Science and Technology, Chiayi 61363, Taiwan; E-Mail: guscsi@gmail.com; 5 Chronic Diseases and Health Promotion Research Center, CGUST, Chiayi 61363, Taiwan; 6 Research Center for Industry of Human Ecology, Chang Gung University of Science and Technology, Taoyuan 333, Taiwan; 7 Department of Pathology, Chang Gung Memorial Hospital at Chiayi, Chiayi 61363, Taiwan; E-Mail: chensm3905@yahoo.com.tw; 8 School of Pharmacy, College of Pharmacy, Kaohsiung Medical University, Kaohsiung 807, Taiwan; 9 Department of Education and Research, Taipei City Hospital, Taipei 112, Taiwan

**Keywords:** Ischemia-reperfusion, endoplasmic reticulum stress, proteomic approach, calretinin

## Abstract

Ischemia-reperfusion injury resulting from arterial occlusion or hypotension in patients leads to tissue hypoxia with glucose deprivation, which causes endoplasmic reticulum (ER) stress and neuronal death. A proteomic approach was used to identify the differentially expressed proteins in the brain of rats following a global ischemic stroke. The mechanisms involved the action in apoptotic and ER stress pathways. Rats were treated with ischemia-reperfusion brain injuries by the bilateral occlusion of the common carotid artery. The cortical neuron proteins from the stroke animal model (SAM) and the control rats were separated using two-dimensional gel electrophoresis (2-DE) to purify and identify the protein profiles. Our results demonstrated that the SAM rats experienced brain cell death in the ischemic core. Fifteen proteins were expressed differentially between the SAM rats and control rats, which were assayed and validated *in vivo* and *in vitro*. Interestingly, the set of differentially expressed, down-regulated proteins included catechol *O*-methyltransferase (COMT) and cathepsin D (CATD), which are implicated in oxidative stress, inflammatory response and apoptosis. After an ischemic stroke, one protein spot, namely the calretinin (CALB2) protein, showed increased expression. It mediated the effects of SAM administration on the apoptotic and ER stress pathways. Our results demonstrate that the ischemic injury of neuronal cells increased cell cytoxicity and apoptosis, which were accompanied by sustained activation of the IRE1-alpha/TRAF2, JNK1/2, and p38 MAPK pathways. Proteomic analysis suggested that the differential expression of CALB2 during a global ischemic stroke could be involved in the mechanisms of ER stress-induced neuronal cell apoptosis, which occurred via IRE1-alpha/TRAF2 complex formation, with activation of JNK1/2 and p38 MAPK. Based on these results, we also provide the molecular evidence supporting the ischemia-reperfusion-related neuronal injury.

## 1. Introduction

Stroke is the fourth-leading cause of death, and ischemic stroke is a major cause of long-term disability in the elderly [1]. Recent advances in understanding the function of the human brain have revealed that the cortical neuron to the infarction plays an important role in the pathobiology of stroke [2]. Reduced blood flow resulting from arterial occlusion or hypotension leads to tissue hypoxia and hypoglycemia. As a result, apoptosis-related mechanisms contribute to the brain damage that occurs after an ischemia-reperfusion injury. The mechanism not only affects the oxidative stress and inflammatory mediators but also alters the permeability of the blood-brain barrier (BBB). This increases the disruption of neuronal-microglial interaction and vulnerability in neuronal cells [3], but the molecular mechanisms need to be demonstrated further [4,5]. Apoptotic cell death includes caspase-dependent and independent pathways [6,7]. Overloaded oxidant and peroxide levels from ischemic stroke and decreased enzymatic antioxidant defenses result in pro-inflammatory markers being found in ischemic-related neuron injury [8,9]. The regulation of neuron apoptosis by oxidative stress has been thoroughly investigated, and oxidative stress has been implicated as the mechanism of neuron injury resulting from ischemic stroke.

The ischemic cascade from ischemia to infarction is caused by arterial occlusion or ischemic stroke. These acute intravascular alterations triggered by the interruption of the blood supply lead to brain damage and subsequent tissue inflammation [10]. Multiple disturbances can induce accumulation of unfolded proteins in the endoplasmic reticulum (ER), triggering the unfolded protein response (UPR). This is known to activate multiple cellular signaling pathways. The activation of IRE1α binds TNF receptor-associated factor 2 (TRAF2), apoptosis signal-regulating kinase 1 (ASK1) and downstream kinases that further activate Jun *N*-terminal kinase (JNK) and nuclear factor-κB (NF-κB) [11]. Reactive oxygen species (ROS) involvement has also been linked to ischemic stroke [12]. Our previous study demonstrated that increased ER stress occurs through p38 MAPK phosphorylation and CHOP protein activation pathways, which may be among the possible pathways involved in stroke-related neuron injury. In addition, we found that increased reactive nitrogen species (RNS) and pro-inflammatory cytokines were released *in vivo* through ischemic administration, which then caused ER stress signaling that has been related to apoptosis by the inducible NOS (iNOS) activation in the neurons [13].

The investigation of the inflammatory process begins in the intravascular compartment immediately after arterial occlusion. This excess oxidative stress induced the apoptosis of neurons by sustained Ca^2+^ release from intracellular stores or by a brief episode of oxygen and glucose deprivation (OGD, *in vitro* model of cerebral ischemia) [14]. Previous studies have reported increased oxidative stress after ischemic injury both *in vivo* and *in vitro* [15]. Apoptosis of neurons resulting from highly reactive oxidants after ischemic insults may be associated with the ER stress pathways, which are activated following transient focal or global cerebral ischemia. We examined the different functions related to the varied protein expression profiles following a model of global ischemic stroke. The proteomic approaches were used to purify and identify protein profiles using 2D differential gel electrophoresis (2D SDS-PAGE) and LC-MS/MS to elucidate differentially displayed proteins.

In this study, we aimed to investigate the molecular mechanisms underlying ischemia-reperfusion injury of the brain-influenced ROS production and the initiation of apoptosis. The OGD paradigm was used in neuron cell cultures to study in more detail the ER stress-related pathways involved in ischemic damage. A gel-based proteomic technique provides substantial information. It concerns both detection of target proteins and valuation of apoptosis and redox imbalance potentials, which involve the ER stress-signaling pathway. Differentially expressed protein spots were identified and analyzed. We had shown reduced expression of catechol *O*-methyltransferase (COMT) and cathepsin D (CATD) protein with increased expression of calretinin (CALB2) protein in the SAM group, through a mechanism involving intracellular ROS. Furthermore, our results showed that OGD induced ER stress by activating IRE1-alpha/TRAF2, JNK1/2, and p38 MAPK pathways. Also, the OGD-mediated activation of CALB2 is a factor of induction dependent on neuron viability and apoptosis via the activation of ER stress-signaling modules.

## 2. Results and Discussion

### 2.1. Cytotoxic Effect of Neuron Cells in Ischemic Rats

**Figure 1:**
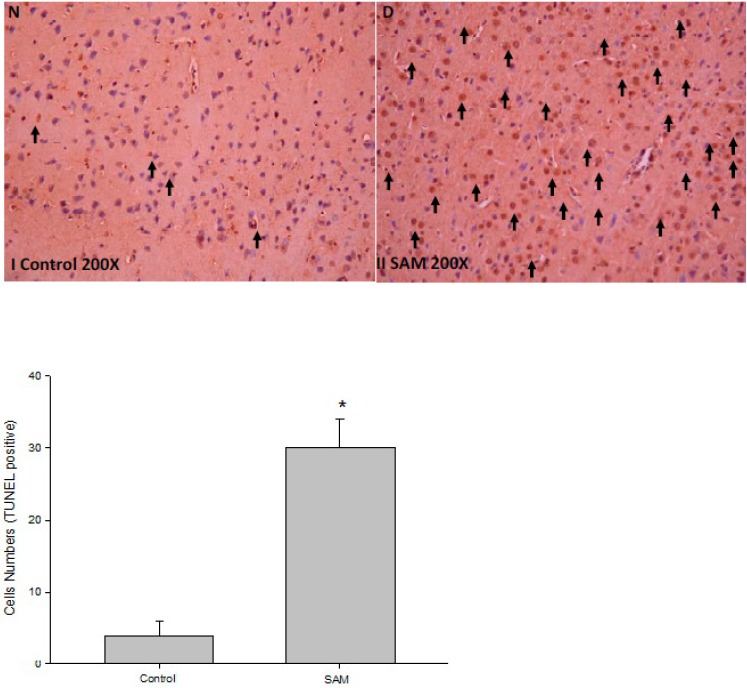
Histological examination of stroke animal model (SAM). Stroke animal model (SAM rat 1.5 h and reperfusion 24 h) were performed on rats as described in “Experimental Section”. Histological examination of brain were revealed to cortex and subcortex zones as indicated by immunohistochemical staining of TUNEL. Representative brain sections stained as control group (I); Rats with transient SAM (II); Apoptotic cells were measured under microscopy as described in Experimental Section. The SAM group exhibited infarct volume attributable to arterial occlusion, as indicated by apoptotic cells (filled arrow). Magnification ×200. * *p* < 0.05, compared with control group.

Our previous investigation showed that a global cerebral ischemic stroke model by temporary ischemia followed with reperfusion onset was accompanied by many complications such as brain impairment, acute inflammation and ROS oxidant in rats. Among these, the most significant complication was progressive brain injury leading to neuron cell death from ischemic lesions in both the cortex and subcortex areas [10]. Based on these studies, we used the TUNEL assay to test whether ischemic stroke affected neuron cell apoptosis in rats. As shown in Figure 1, global immunoreactivity to apoptosis was localized primarily within the total brain area (*p* < 0.05, *n* = 6) for both cortical and subcortical infarctions in SAM rats. Quantitative examination of the brain pathology showed that the number of positive TUNEL neurons present in the SAM group was higher than the number of neurons present in the control group (control group = 4 ± 4; SAM group = 31 ± 5, *p* < 0.05).

### 2.2. Identification of Differentially Expressed Proteins after Ischemia Reperfusion Brain Injury

**Figure 2:**
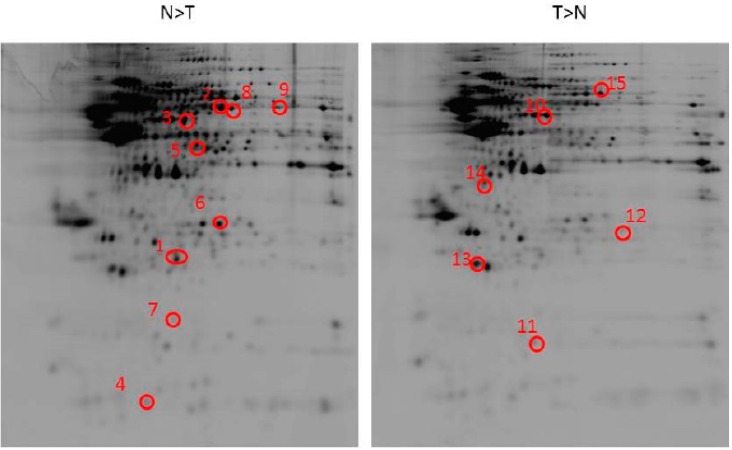
Representative two-dimensional gel electrophoretograms of brain section protein in control and transient stroke animal model (SAM) rats. Rats were sacrificed and the brains were separated. Representative brain sections stained as control group (I); rats with transient SAM (II). 250 μg of brain was subjected to IEF (pH 3–10), SDS-polyacrylamide gel separation and silver staining. The number in the red circles indicate the each protein spots. Six pairs of brain were used, and a representative pair of the proteomic gel images is shown. Fifteen protein spots with a three-fold difference between both groups were subjected to MALDI-TOF-TOF analysis. Nine spots with a significant decrease in SAM group were enlarged and elucidated as numbers. Six spots with a significant increase in SAM group were reported. The abbreviated names of these differentially displayed proteins are listed in Table 1.

**Figure 3:**
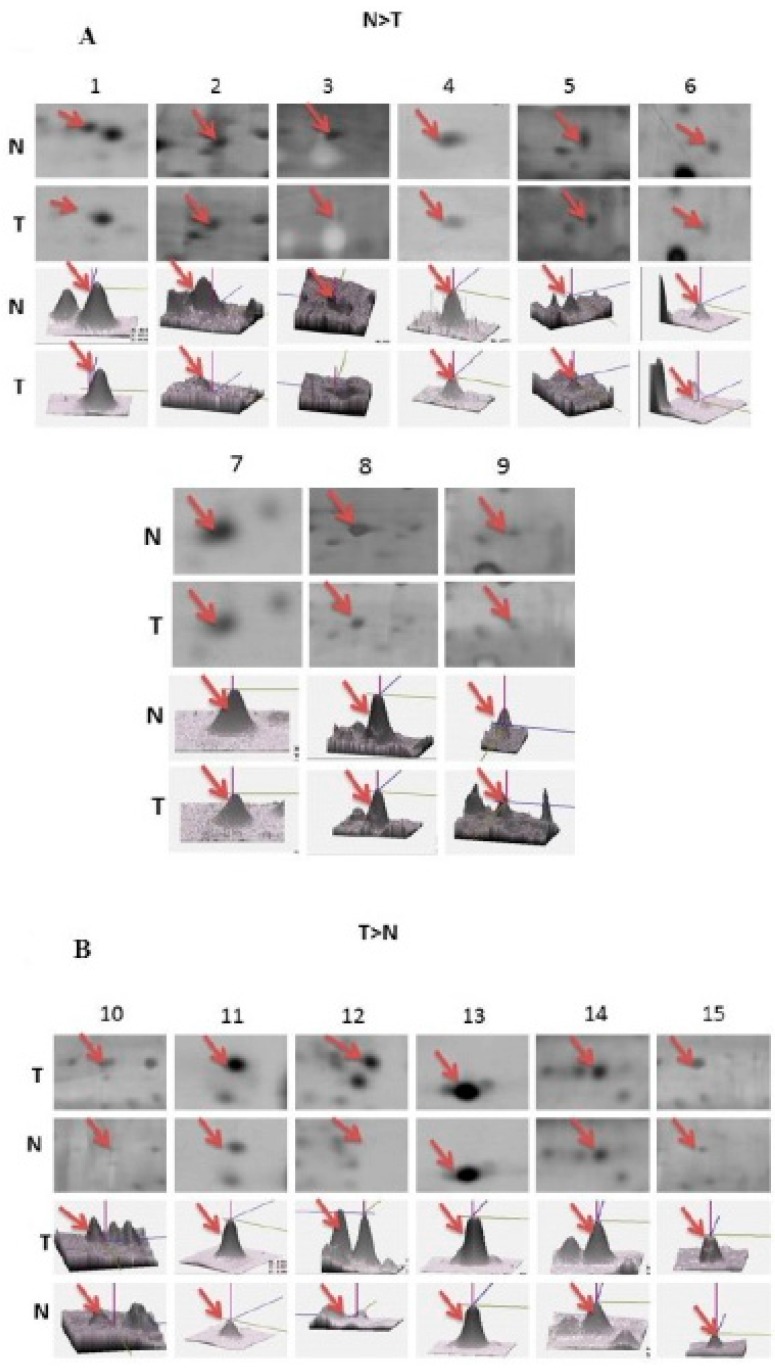
A close view of selected protein spots representing differentially expressed SAM-related proteins between in control and SAM-operated rats. The proteins with expression compared with SAM group are shown. (**A**) Nine proteins were consistently down-regulated; (**B**) Six identified proteins showed a greater-than-threefold change in density. The red arrows correspond to that in Table 1. Six pairs of brain protein extracts were evaluated, and the results shown are derived from one pair of experiments; fifteen reproducible blots were performed in total.

The proteomic profile of the cortex region was used to examine the events of SAM-induced brain infarction and stress responses. In this study, the protein of the cell was extracted from the control group and SAM-treated samples (*n* = 6 for each group). Protein samples were prepared, and more than 400 protein spots were viewed. Using silver-staining and 2D-PAGE analysis, several differences were observed with transient global ischemia in the six pairs of rats. Protein expression gel profiles (six pairs from both rat groups) were compared using ImageMaster software (Canon A640, Tokyo, Japan). The focus was then on identifying proteins found to be statistically significant at more than threefold after ischemic injury. When proteins were resolved by SDS-PAGE electrophoresis on a 12% gel, protein spots appeared to be differentially expressed more than three times greater in the SAM group than in the control group (Figure 2). In SAM rats, nine proteins were consistently down-regulated (Figure 3A), while six identified proteins showed a greater-than-threefold change in density (Figure 3B). All spots were subjected to peptide fingerprint identification using MALDI-TOF/TOF. Fifteen proteins were successfully identified by MALDI-TOF/TOF Mass Spectrometry (Table 1). Zoomed views of representative gel regions depicted several differentially expressed proteins related to oxidative stress, inflammatory response and apoptosis-related proteins. Statistical analyses were conducted after quantification of the differentially expressed proteins.

**Table 1:** Fifteen proteins were successfully identified by MALDI-TOF/TOF Mass Spectrometry.

Spot	Protein Name	*Mr*/*PI*	Accession No.	MASCOT Score	Matched Peptides
1.	Catechol *O*-methyltransferase	29/5.3	COMT_RAT	637	47
2.	Protein disulfide-isomerase A3	56/8.8	PDIA3_RAT	108	21
3.	Guanine deaminase	50/6.0	GUAD_RAT	58	15
4.	Cystic fibrosis transmembrane conductance regulator	167/9.5	CFTR_RAT	89	10
5.	Cathepsin D	44/6.8	CATD_RAT	375	21
6.	Guanidinoacetate *N*-methyltransferase	26/5.6	GAMT_RAT	437	30
7.	d-dopachrome decarboxylase	13/6.1	DOPD_RAT	332	27
9.	d-3-phosphoglycerate dehydrogenase	26/5.6	SERA_RAT	437	30
10.	Dihydropyrimidinase-related protein 2	62/6.4	DPYL2_RAT	59	14
11.	Histidine triad nucleotide-binding protein 1	13/5.5	HINT1_RAT	63	9
12.	Protein-l-isoaspartate (d-aspartate)*O*-methyltransferase	24/7.8	PIMT_RAT	483	27
13.	Beta-synuclein	14/4.3	SYUB_RAT	714	105
14.	Calretinin	31/9.6	CALB2_RAT	73	13
15.	Neurexin-3-alpha	173/6.1	NRX3A_RAT	94	16

### 2.3. Validation of Differentially Displayed Proteins on Brain Neuron Histopathological Changes

**Figure 4:**
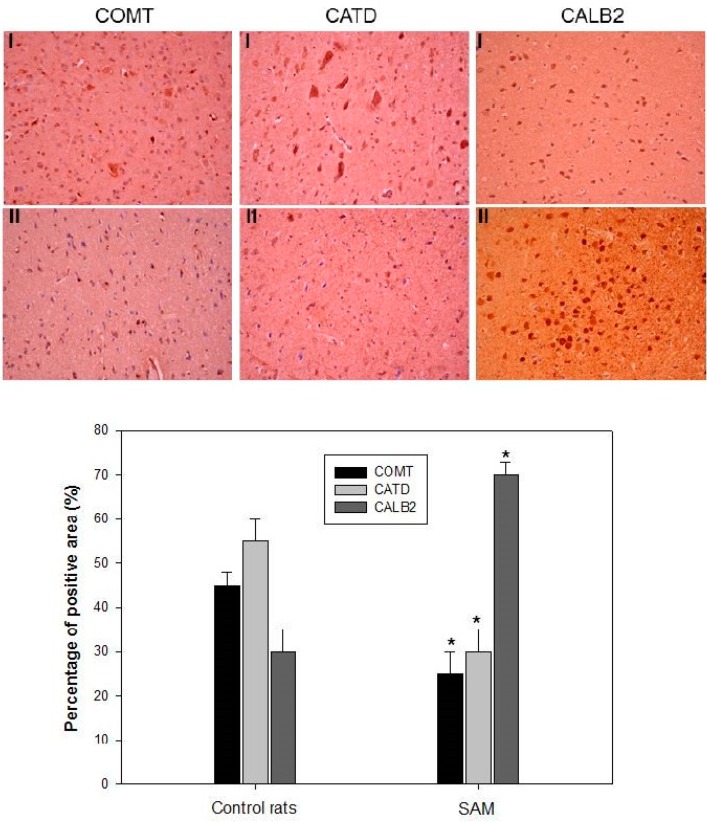
Immunohistochemical staining for indicated proteins expression in rat brains was analyzed: Saline infusion control rats (I); rats with SAM (II). The positive stained area was evaluated from three randomly selected observation fields of each brain sections. Quantitative of immunohistochemical proteins COMT, CATD and CALB2 by average integrated optical density (AIOD). Positive stained area was evaluated from three randomly selected observation fields of each brain section. Data were expressed as mean ± SD (*n* = 6/group). * *p* < 0.05, compared with control group. Magnification ×400.

Spots 1, 5 and 14 were subsequently identified using 2D proteomic analysis, including Catechol O-methyltransferase (COMT), Cathepsin D (CATD), as well as Calretinin (CALB2), which may mediate the oxidative injury effects in the neuronal cells of rats with ischemic stroke (Table 1). In order to verify the effects of intravenous SAM administration on the relationships between neurotoxicity, inflammation and oxidative stress in the neuron cells were examined by using 2-DE proteomic analyses. These proteins were selected for further examination using immunohistochemistry assays *in vivo* after ischemic injury and OGD treatment in N2a cells *in vitro*. As shown in Figure 4, rats with ischemic impairment demonstrated reduced expression of COMT and CATD in the brain tissue samples. In particular, ischemic brain damage resulted in significant expression of CALB2. These results are consistent with the proteomic results, indicating that a proteomic differential display model is applicable when assessing SAM-induced brain infarction.

### 2.4. Effects of OGD Treatment on the Up-Regulation of CALB2, the ER Stress-Signaling Mechanisms and on ROS-Related Apoptosis

**Figure 5:**
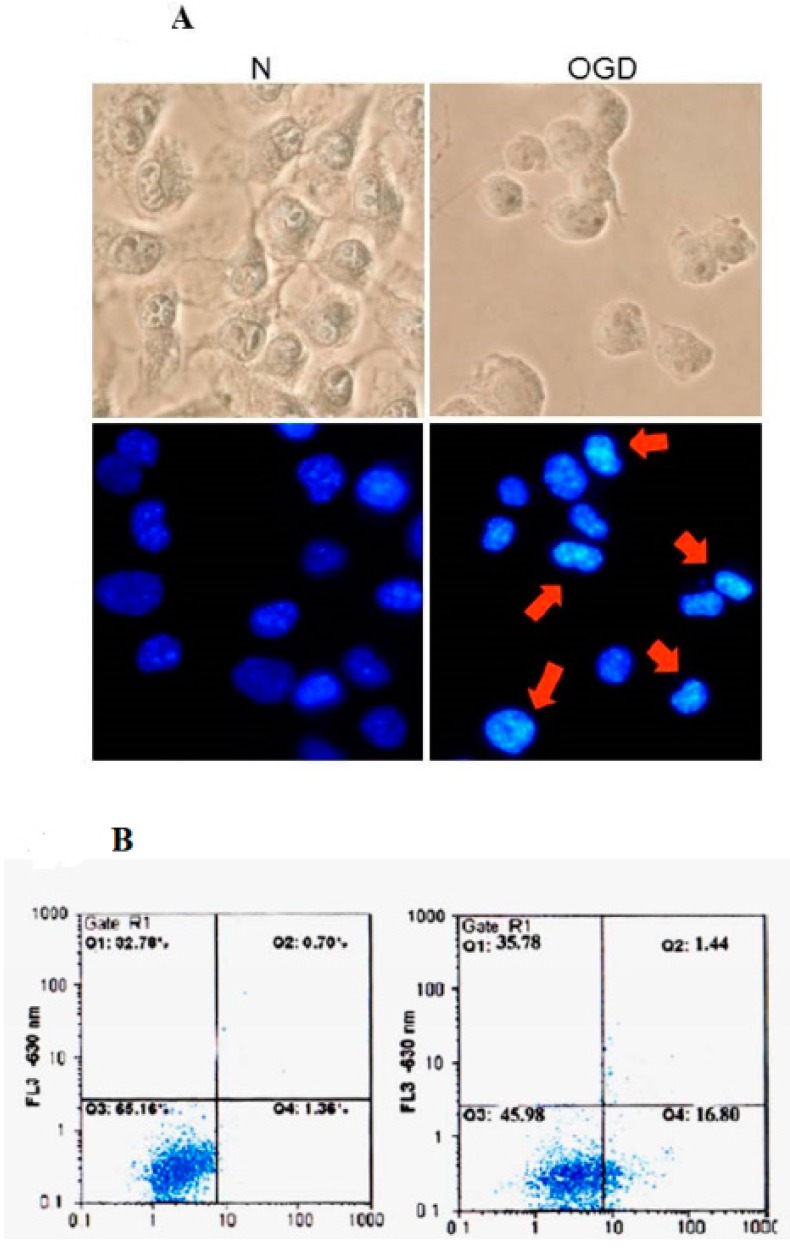
Effects of oxygen-glucose deprivation (OGD) on morphological characteristics, and assessment of cell death in Neuro-2a (N2a) cells. (**A**) Changes in nuclei by DAPI staining. N2a cells were treated with OGD, and stained with DAPI. Apoptotic cells were measured under fluorescence microscopy as described in Materials and methods. Apoptotic cells indicate condensed and fragmented nuclei (red arrows). Magnification, ×200; (**B**) After an indicated treatment for 24 h, the cells were stained with FITC-conjugated Annexin-V and PI for flow cytometry analysis as described in Materials and Methods. The percentages presented in each frame depicted the apoptotic cells.

It was reported in a previous study that the deprivation of oxygen and glucose (OGD) in the ischemic brain eventually leads to cell death. Accumulation of unfolded protein response and oxidative stress were believed to be the main contributors to neuron death and ER stress-inducing involvement of the signaling pathway by apoptosis [14,16]. It was necessary to determine whether the OGD model of neuronal ischemia is cytotoxic to human N2a cells and to the molecular mechanisms underlying the OGD-mediated activation of IRE1-alpha inducing Jun *N*-terminal kinase (JNK1/2) and p38 MAP Kinase (MAPK). The MTT assays were performed after N2a cells were exposed to an ischemic environment for 24 h to examine the protein levels under regulation of ER stress. As shown in Figure 5, exposure to OGD for 24 h caused N2a cells to show the characteristic features of cell shrinking, rounding, and apoptotic body formation. The extent of apoptosis of OGD induction was quantified as a percentage of annexin V-positive cells and was shown as 18%. OGD treatment of human N2a cells also resulted in decreased levels of COMT and CATD, while OGD induced CALB2 expression (Figure 5). We therefore found that, compared to the control group, elicitation with OGD significantly increased total lysate protein CALB2 and decreased COMT and CATD. In addition, endogenous IRE1-alpha co-immunoprecipitated with TRAF2 in N2a cells induced after two and three hours of OGD doses. Taken together, previous results showed that increased ROS production induced by OGD [14] is often ER stress-associated with the up-regulation of IRE1-alpha/TRAF2, JNK1/2, and p38 MAPK pathways as a mechanism increasing ischemic injury [17,18]. We detected one protein, CALB2, a calcium-binding protein, which previously had been coordinated in the endoplasmic reticulum-mitochondria and the intrinsic apoptotic pathway [19]. To further examine OGD-induced damage in N2a cells, we studied the effects of kinase inhibitor as it gave resistance to OGD-induced apoptosis and oxidative damage. As shown in Table 2, N2a cells were followed to OGD and then co-treated with inhibitors, p38/MAPK inhibitor SB203580, JNK1/2 MAPK inhibitor SP600125 as well as the ROS scavenger NAC groups. This treatment significantly reversed the OGD-induced apoptosis to 21%, 10%, 8% and 5%, respectively (*p* < 0.01) and then increased SOD activity of neuron cells to 35%, 77%, 80% and 88%, respectively (*p* < 0.01); however, ERK1/2 MAPK inhibitor had no effect.

**Table 2:** Effects of the kinase inhibitor on the OGD induction associated with apoptosis and oxidative status in N2a cells.

	% of Cell Death	SOD Activity (%)
Control	2	100
OGD	21	35
OGD-SB	10	77
OGD-SP	8	80
OGD-NAC	5	88

### 2.5. ROS, IRE1-alpha/TRAF2, JNK1/2/, and p38 MAPK Triggering Pathways Are Involved in the Regulation of OGD-Induced CALB2 Expression in Neuron Cells

**Figure 6:**
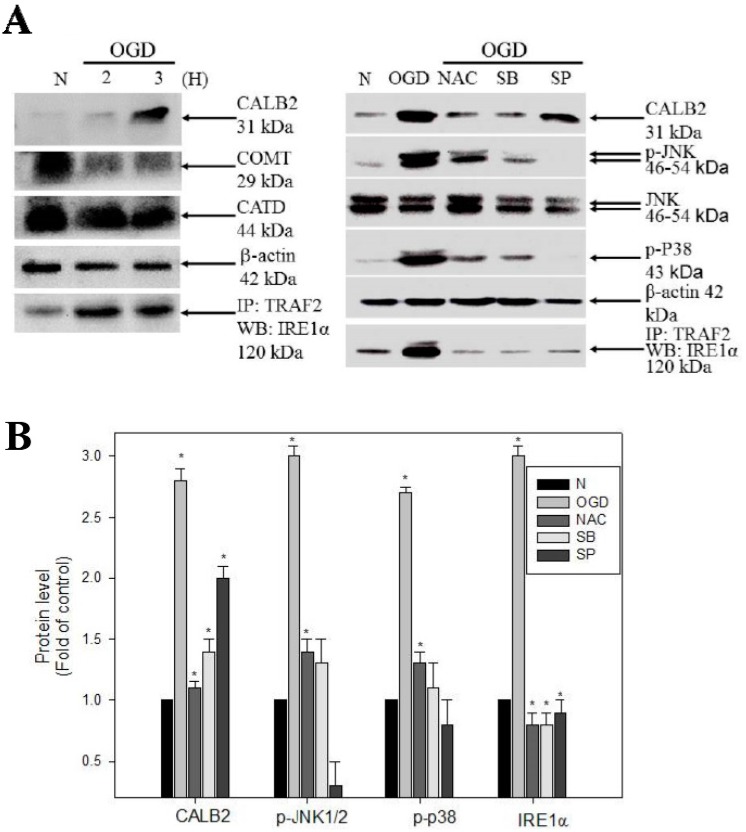
Immunohistochemistry staining for indicated proteins in rat brains. Validation of the identified differential proteins and oxygen-glucose deprivation (OGD)-associated molecules between untreated (Control) and treated (OGD) in N2a cells. (**A**) Data presented in western blots are derived from a representative study, and comparisons of protein expression are calculated from three replicate experiments. The association of TRAF2 with IRE1-a was determined by immunoprecipitation with TRAF2 followed by western blot with anti-IRE1-alpha antibody; (**B**) Effects of the kinase inhibitors blocking OGD-induced expression of ER stress related proteins. Kinase inhibitors were treated with or without OGD after 1 h. Total cell lysates were prepared and subjected to western blot analysis. Protein levels of phosphorylated JNK1/2 and p38 MAPK and the association of TRAF2 with IRE1-alpha and β-actin were detected with the indicated antibodies. * *p* < 0.05, compared with control group.

Our results clearly demonstrated that OGD resulted in neuron cell death and that oxidative damage was relative to the expression of CALB2 and activation of ER stress-signaling pathways. Recent studies have indicated that regulation of excess calcium release results from oxidative stress, and many calcium-binding proteins, such as CALB2, may function downstream of the ER calcium release to modulate apoptosis in response to neuronal damage [20]. To investigate the role of CALB2 in OGD-mediated oxidative impairment and ER stress signaling, a basic study was conducted to demonstrate the novel role of CALB2 by a brief event of OGD. As shown in Figure 6, NAC almost blocked the OGD-elicited expression of CALB2 as well as the phosphorylation of JNK1/2 and p38 MAPK and the interaction of IRE1-alpha/TRAF2 complex, as compared to the OGD model of 1 h. In addition, OGD co-treated with p38/MAPK inhibitor SB203580 resulted in the inhibition of the OGD-induced expression of the CALB2 and IRE1-alpha/TRAF2 complex. At the same time, JNK1/2 MAPK inhibitor SP600125 reduced the effects on the IRE1-alpha/TRAF2 association (Figure 6). Taken together, the results showed that up-regulation of CALB2 was essentially involved in oxidative stress-signaling OGD-influenced cell death with both IRE1-alpha/TRAF2 and JNK1/2 and p38 MAPK-related pathways. The results confirmed that OGD-treated ischemic injury was associated with the IRE1-alpha/TRAF2, JNK1/2, and p38 MAPK-dependent signal transduction and increases in CALB2 expression.

Biophysical and biochemical ischemic damage resulted when blood flow disarray, brain edema, hemorrhagic stroke, thrombosis clot and glucose deprivation occurred. Accumulation of generated ROS, impaired intracellular calcium turnover, secretion of interleukins and prostaglandins, are triggered by ischemic damage and may lead to brain dysfunction and neuronal cell death while the oxygen and glucose delivery via blood flow in the brain is interrupted [21]. It is well-known that neurons are more susceptible than glia and vascular cells to such changes in the ischemic brain environment [22]. In this study, we demonstrated an increase in SOD depletion and apoptosis initiation by ROS oxidants in the brain of SAM-treated rat and OGD-induced N2a cells (Figures 1 and Figure 5, Table 2). Quantification of pathological neuron cells showed that the number of normal neurons was significantly reduced in ischemic impairment (Figure 1). However, the effects of inflammatory and oxidative stress are still not fully understood with respect to the biological processes responsible for the targeting of ischemic-related protein profiles and the progression of neuronal injury and brain damage. Understanding these mechanisms, especially after brain injury, is necessary to completely explain the long-term effects of ischemic brain injury and to help the development of new practical therapies for stroke.

Despite the intense efforts of developing efficient treatment for ischemic stroke, 2D gel-based proteomic analysis also plays a significant role by identifying proteins potentially involved in the mechanisms of ischemic brain damage (Figure 2). Furthermore, the proteomic analysis of the disease state allows investigation of the molecular mechanisms of disease pathogenesis. This showed the significant differences in protein expression profiles in rat brain neurons as an insult of ischemic stroke. Our proteomics results showed that catechol O-methyltransferase (COMT) and cathepsin D (CATD) were more insufficient in ischemic damage, while calretinin (CALB2) was abundant in brain injury (Figure 3). Recent studies have suggested that a partial COMT absence in rats has been found to induce cell death with the inactivation of dopamine, norepinephrine, epinephrine, caffeine, estrogen, and catechol compounds by distribution of the cortical neurons, with specific involvement of the modulation in cortical dopamine signaling such as dramatic evolutionary changes in cognitive function [23]. Hence, it is sensible to suggest that patients use modified COMT expression as an alternative-medicine strategy. The Cathepsin D (CATD) deficiency, lysosomal aspartic protease storage, commonly expressed by neurondegenerative disease in the porousness of the BBB and infiltration of peripheral blood mononuclear cells, significantly increased oxidative damage in mice as an acute response to neuropathogenesis [24]. Our results indicate that during cerebral ischemia, a large decrease in the expression of COMT and CATD occurs in the brain tissue (Figure 4), probably as a result of inflammatory and oxidative failure of the neuron cells’ death. This leakage of dopamine or neurotransmitters may be a causal factor in the neuronal damage associated with cerebral ischemia [25].

**Figure 7:**
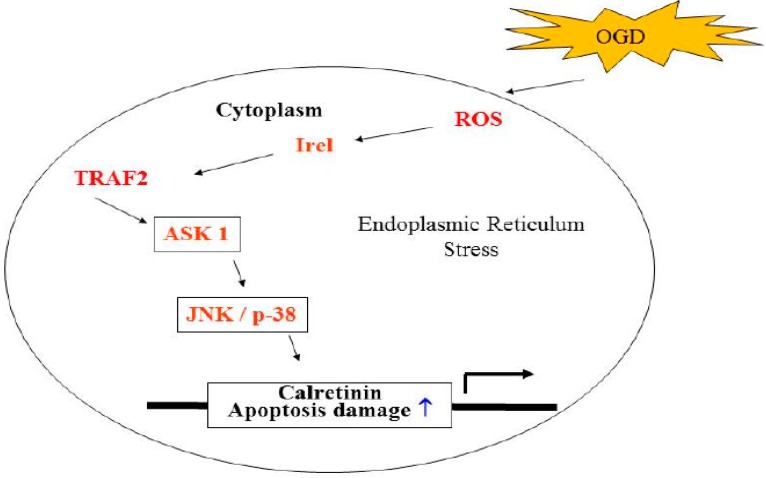
Schematic presentation of the signaling pathways involved in OGD-induced cells apoptosis in N2a cells. The effect of ischemic stroke on the ER stress triggering production of ROS activates IRE1-alpha/TRAF2 complex formation and phosphorylation of JNK1/2 and p38 MAPK pathways, which induces the calretinin up-regulation and increases the apoptosis cascade.

The induction of apoptosis in neuronal cells after ischemic stroke is a critical feature of oxidative stress [22]. Oxidative stress occurs when the generation of reactive oxygen species (ROS) or their products with excessive uncontrolled antioxidant protection, contributes to the neurodegenerative disorders and the pathological changes in ischemic brain injury. Ischemia reperfusion impedes BBB effects on oxygen consumption and glucose in neuronal morphology and function, including the survival and the plasticity of the neurons. These factors accumulate the peroxidative damage of particular proteins’ reactions, which are important regulators of intracellular signaling [21,22]. In this study, we demonstrated the target of stroke injury. Increased expression in CALB2, along with apoptosis initiated via the activation of JNK1/2, p38 MAPK signaling and ROS, triggers modules in OGD-treated N2a neuron cells (Figure 5, Table 2). Interestingly, CALB2, a calcium-binding or calcium-buffering protein that has been used as a distinguishing marker destined to be modified in ER, functions in maintaining calcium hemostasis and in leading neuron apoptosis injury. Among Ca^2+^ binding protein, calretinin is believed to play an important role in calcium homeostasis. Calretinin (CALB2) is a Ca^2+^ binding protein present in various populations of neurons distributed in the central and peripheral nervous systems. Previous study of deficient (−/−) mice has shown that regulation of the [Ca^2+^] ion plays a crucial role in information processing in the cerebellar cortex and is involved in the induction of synaptic plasticity [12]. To validate these findings, further study using CALB2 ablation is needed to determine whether there are mediated actions to induce ischemic stroke neuron injury in calretinin knock-out mice [26,27]. Many studies have shown that lower calcium can promote apoptotic injury in neurons, while ROS can target ER resident proteins, chaperones and calcium channels, leading to the release of calcium from the ER into the cytosol and ER-stress signaling to correlate with increased neuronal death [19,20,21]. In addition, we suggest that these activation effects result from a downstream gene of IRE1α/TRAF2 illustration after the OGD periods and that these effects occur in response to ER stress signaling (Figure 6). It has been shown previously that the activation of c-Jun *N*-terminal kinases (JNK) and p38 mitogen-activated protein kinase (p38/MAPK), also called stress-activated protein kinases, has been implicated in many cellular processes such as the neuronal death associated with cerebral ischemia [17,18]. Our results showed that OGD expression of differentially displayed CALB2 proteins had an effect on the cytotoxicity of neuron cells by activating the JNK1/2 and p38 MAPK pathway, which is preceded by the association of IRE1α/TRAF2, an induction of ROS (Figure 7).

Taken together, differentially displayed proteins were assayed in the rat brain cells and validated *in vivo*. We used neuron protein from OGD treatment to evaluate the mechanism involved in the induction of ER stress and oxidative stress [16,28]. These proteins point to ischemic reperfusion injury and the targeting of oxidative stress using proteomic analysis. The role of oxidative stress in stroke and stroke pathogenesis has been well described. However, little is known with regard to the contribution of differentially displayed proteins to neurodegenerative disorders and ischemic stroke [29,30]. Studies in the near future, by our laboratory and others, will identify whether oxidative stress in ischemic brain injury plays an essential role in propagating neurodegenerative disorders; the details of this function are needed for further analyses. However, further studies of many natural products that we consume daily and studies of Chinese herbs, considered as suppressing or protective agents, are still required to validate these findings relating to antioxidant-based stroke intervention via brain-targeted antioxidants.

## 3. Experimental Section

### 3.1. Induction of Ischemia Reperfusion Brain Injury

Adult male Sprague-Dawley rats weighing around 280 ± 20 g were kept individually in a 12-h light/dark cycle cage and had free access to water and food. Animal care and the general protocols for animal use were approved by the Institutional Animal Care and Use Committee of National Yang-Ming University. All efforts were made to minimize the number of animals used and their suffering. These rats were operated according to the modified global cerebral ischemia’s model, by two vessel occlusion (2VO) to induce reversible ischemia for a limited time period as described in our previous study [31]. In brief, the bilateral common carotid arteries were ligated with 4-0 nylon under anesthetized by 10% chloral hydrate (350 mg/kg, injection with 1.0 mL of the solution). These filaments were withdrawn 90 min after ligation which ischemia was achieved. The femoral artery and vein were exposed and cannulated with PE-50 polyethylene tubing (Fisher Scientific, Fair Lawn, NJ, USA) [13]. Sham-operated control animals underwent all the surgical procedure except the arteries were not ligated. Two groups (six rats in each group) were randomly assigned to a sham control group or a stroke group.

### 3.2. Chemicals and Reagents

H_2_DCFDA (2,7-dichlorodihydrofluorescein diacetate) and anti-β-actin were purchased from Sigma (St. Louis, MO, USA). *O*-methyltransferase (COMT), cathepsin D (CATD), calretinin (CALB2) and horseradish peroxidase-linked anti-rabbit or anti-mouse IgG were obtained from Santa Cruz Biotechnology (Santa Cruz, CA, USA). Anti-caspase 9, anti-phospho-p44/42 MAPK, anti-phospho-JNK1/2, CHOP and horseradish peroxidase-linked anti-rabbit or mouse IgG were obtained from Cell Signaling Technology, Inc. (Beverly, MA, USA). Monoclonal anti-CPP32 to detect caspase 3, inositol-requiring kinase 1 (IRE1), NFκB p50, Bcl-2, suppressor of cytokine signaling (SOCS) and TNF receptor-associated factor 2(TRAF2) were obtained from Santa Cruz Biotechnology (Santa Cruz, CA, USA). Janus kinase (JNK) inhibitor (SP600125), and p38 inhibitor (SB203580) were purchased from Sigma (St. Louis, MO, USA). Rabbit monoclonal antibodies against phospho-p38 MAPK (Thr180/Tyr182) and phospho-JNK1/2 (Thr183/Tyr185) and mouse monoclonal antibody against Phospho-Akt (Ser473) were purchased from Cell Signaling Technology (Beverly, MA, USA). The TdT-mediated dUTP Nick End Labeling (TUNEL) kits were purchased from Roche (Mannheim, Germany). SDS, NP-40, while sodium deoxycholate, protease inhibitor cocktail was purchased from Sigma (St. Louis, MO, USA) [32].

### 3.3. Analysis of Brain Proteomic Profiles in Transient Ischemia Rats Using Two-Dimensional Protein Electrophoresis

The brain was cut into seven 2-mm coronal slices using a rat brain matrix and the brain slices were used for cellular lysates preparation as described in our previous study [13]. Cellular lysates were prepared by suspending brain sections and the protein content in the supernatant was quantified using a BCA protein quantitation assay and then subjected to immunoblotting using Immobilon-P membranes (Millipore, Bedford, MA, USA) as the indicated secondary antibodies. Signals were detected using an enhanced chemiluminescence Western blot kit as described previously [33,34]. The chemicals and reagents used for 2D gel electrophoresis were as described in our previous study [35]. Brain proteins in control group and stroke group rats were harvested. Prior to 2D-PAGE analysis, protein concentrations were measured using the Bradford assay with bovine serum albumin as the standard sample for normalization. Serum proteins were precipitated using 10% trichloroacetate in acetone. Brain proteins from coronal slices (250 μg) were suspended in rehydration solution and subjected to isoelectric focusing (IEF) in 13-cm, nonlinear, pH 3–10, immobilized-gradient strips (ImmobilineDryStrips, Amersham Biosciences, Uppsala, Sweden) in an Ettan IPGphor II apparatus (Amersham Biosciences). The second dimension electrophoresis was carried out using 10% SDS-PAGE gels. Gels were fixed and subjected to silver staining, and the reaction was finally stopped with 3.65 g EDTA in 250 mL ddH_2_O.

### 3.4. In-Gel Digestion and Identification of Peptide Fingerprints with Serum Proteins Using MALDI-TOF

There were in total twelve rats in this study, with six in the stroke animal model (SAM) group, and six rats in the control group by randomized selection. Six individual pairs of silver-stained 2D SDS-PAGE gels in which plasma proteins between SAM and control group had been resolved and scanned using ImageMaster 2D Platinum Software 6.0 (Amersham Biosciences). The protein profiles were recorded and compared as previously described [36]. Only the protein spots that appeared and differed by at least 3-fold in all six pairs of 2D gels were subjected to in-gel digestion for matrix-assisted laser desorption/ionization-time-of-flight/time-of-flight (MALDI-TOF/TOF) mass spectrometric analysis. The gel pieces were then dehydrated and subjected to trypsin digestion. Mass spectra were acquired as the sum of the ion signals generated by target irradiation with a mean of 300 laser pulses using the FlexAnalysis system (Bruker-Franzen Analytik, Bremen, Germany). Peptide fingerprints were selected in the mass range of 700 to 4000 Daltons and were analyzed using the Mascot software package. A Mascot score with *p* < 0.05 was considered statistically significant as described in our previous study. MALDI-TOF/TOF data were searched in-house using the Mascot software package (ver. 2.2.04). Protein identification required detection of unique peptides. Proteins with more than two spectral counts were selected for further analysis. The peptide mass data corresponding to each spot were searched against the SwissProt human database using Mascot search engines.

### 3.5. Immunohistochemistry

Immunohistochemistry (IHC) staining was performed using a biotinylated secondary antibody (Vectastain Universal Elite ABC Kit, Burlingame, CA, USA). Monoclonal rabbit antibodies against peroxiredoxin2 and alpha-1-antiproteinase were diluted at a ratio of 1:100. The omission of primary antibodies was used as the negative control. For three slides, cytoplasm that was stained brown was scored as positive. The expression of COMT, CATD and CALB2 were quantitatively evaluated using an Olympus CX31 microscope (Tokyo, Japan) with the Image-pro Plus medical image analysis system. Digital images were captured using a digital camera (Canon A640, Tokyo, Japan). The positive area and optical density (OD) of COMT, CATD and CALB2 positive cells were determined by measuring three randomly selected microscopic fields (400× magnification) for each slide. The IHC index was defined as the average integral optical density (AIOD) (AIOD = positive area × OD/total area) [37].

### 3.6. Oxygen Glucose Deprivation (OGD) of Ischemia

The N2a (Neuro-2a) mouse cell line was purchased from the American Tissue Culture Collection (ATCC, Manassas, VA, USA) and was grown in Dulbecco’s Modified Eagle Medium (DMEM) (Gibco, Invitrogen, Carlsbad, CA, USA). N2a was incubated with DMEM containing 10% fetal bovine serum (FBS) and 1% penicillin/streptomycin in a humidified atmosphere of 5% CO_2_ and 95% air at 37 °C for controls. OGD was induced by incubating neurons in this medium and placing neurons in an anaerobic chamber at 37 °C in 95% N_2_ and 5% CO_2_ glucose-free DMEM in a HEPES-buffered glucose-free medium. OGD was treated after 2 or 3 h by exchanging the glucose-free medium back to the normal cell culture incubator for 22 h duration of re-oxygenation (reperfusion) [14].

### 3.7. Statistical Analyses

The experiments were performed as triplicate independent experiments, and data were presented as three repeats from one independent experiment. Data are reported as the mean ± standard deviation and evaluated by one-way ANOVA. SPSS version 16.0 (SPSS, Inc., Chicago, IL, USA) was used for all statistical analyses. Significant differences were established at *p* < 0.05 [34].

## 4. Conclusions

Oxidative stress is implicated in ischemia reperfusion-related stroke in animal models, and neuron apoptosis is also observed in OGD induction [5]. COMT and CATD have shown significant reductions in expression in the ischemic brain using a two-dimensional electrophoresis-based proteomic analysis. Protein expression of CALB is also significantly induced after ischemic stroke-induced brain injury (Figure 7). Further studies are required to establish the functional roles of identified proteins in stroke-related brain damage and also to determine their roles in peroxidation and ER-stress signaling in neuronal cell pathology.
